# Distinctly altered gut microbiota in the progression of liver disease

**DOI:** 10.18632/oncotarget.8466

**Published:** 2016-03-29

**Authors:** Guoxiang Xie, Xiaoning Wang, Ping Liu, Runmin Wei, Wenlian Chen, Cynthia Rajani, Brenda Y. Hernandez, Rosanna Alegado, Bing Dong, Defa Li, Wei Jia

**Affiliations:** ^1^ Shanghai Key Laboratory of Diabetes Mellitus and Center for Translational Medicine, Shanghai Jiao Tong University Affiliated Sixth People's Hospital, Shanghai, China; ^2^ University of Hawaii Cancer Center, Honolulu, Hawaii, USA; ^3^ E-institute of Shanghai Municipal Education Committee, Shanghai University of Traditional Chinese Medicine, Shanghai, China; ^4^ Key Laboratory of Liver and Kidney Diseases (Ministry of Education), Institute of Liver Diseases, Shuguang Hospital, Shanghai University of Traditional Chinese Medicine, Shanghai, China; ^5^ Department of Oceanography, University of Hawaii at Manoa, Honolulu, Hawaii, USA; ^6^ National Key Laboratory of Animal Nutrition, China Agricultural University, Beijing, China

**Keywords:** gut microbiota, lipopolysaccharides, liver disease, pathogenesis, Immunology and Microbiology Section, Immune response, Immunity

## Abstract

Recent studies underscore important roles of intestinal microbiota and the bacterial lipopolysaccharides (LPS) production in the pathogenesis of liver disease. However, how gut microbiota alters in response to the development of steatosis and subsequent progression to nonalcoholic steatohepatitis (NASH) and hepatocellular carcinoma (HCC) remains unclear. We aimed to study the gut microbial changes over liver disease progression using a streptozotocin-high fat diet (STZ-HFD) induced NASH-HCC C57BL/6J mouse model that is highly relevant to human liver disease. The fecal microbiota at various liver pathological stages was analyzed by 16S rDNA gene pyrosequencing. Both UniFrac analysis and partial least squares-discriminant analysis showed significant structural alterations in gut microbiota during the development of liver disease. Co-abundance network analysis highlighted relationships between genera. Spearman correlation analysis revealed that the bacterial species, *Atopobium spp.*, *Bacteroides spp., Bacteroides vulgatus, Bacteroides acidifaciens*, *Bacteroides uniformis, Clostridium cocleatum, Clostridium xylanolyticum* and *Desulfovibrio spp.,* markedly increased in model mice, were positively correlated with LPS levels and pathophysiological features. Taken together, the results showed that the gut microbiota was altered significantly in the progression of liver disease. The connection between the gut microbial ecology and the liver pathology may represent potential targets for the prevention and treatment of chronic liver disease and HCC.

## INTRODUCTION

Chronic liver disease, one of the major causes of morbidity and mortality [1], is a highly dynamic process leading to liver fibrosis and cirrhosis, and eventually, hepatocellular carcinoma (HCC). The liver interacts directly with the gut through the hepatic portal and bile secretion systems. The intestinal epithelium, along with its colonizing bacteria, represents a first site of interactions between diet and the host immune system. This interaction can impact on the structure and composition of the gut microbiota [2], which in turn directly affects the gut-immune homeostasis and intestinal permeability [3]. Imbalance of the gut microbiota is associated with a number of hepatic diseases from lipid accumulation in hepatocytes to stellate cell activation, immune cell recruitment, and cancer development [4]. There is growing evidence that the pathophysiology and treatment of a wide array of liver diseases are likely to be strongly influenced by the nature and/or manipulation of gut microbiota [5]. Disruption of microbial and intestinal homeostasis is associated to the most prevalent chronic liver diseases: non-alcoholic fatty liver disease (NAFLD)/nonalcoholic steatohepatitis (NASH), and alcoholic liver disease (ALD) [6, 7]. A recent study indicates that germ-free mice colonized with intestinal microbiota from mice with high fat induced hyperglycaemia developed hyperglycaemia. In contrast, mice colonized with intestinal microbiota from the mice with normoglycaemia did not [8]. Studies also suggest that germ-free mice are more susceptible to experimental liver fibrosis [9]. Studies have also demonstrated that intestinal inoculation with a single bacterium, *Helicobacter hepaticus*, can disrupt enterohepatic homeostasis and promote liver cancer [10].

Gut microbiota dysbiosis, especially the microbial translocation and their products such as endotoxin (lipopolysaccharides, LPS) across the intestinal gut barrier is common in patients with chronic liver diseases [11–13]. LPS, a major component of the outer membrane of Gram-negative bacteria [14], has been demonstrated an early factor in the triggering of high-fat diet (HFD)-induced metabolic diseases [15]. For instance, HFD intake has been shown to be associated with elevated portal and systemic circulating levels of LPS [15]. Germ-free mice colonized with one LPS-producing bacterium, *Enterobacter cloacae* B29 isolated from a morbidly obese human's gut induced obesity and insulin resistance with HFD, whereas the germ-free control mice on a HFD did not exhibit the same disease phenotypes [16]. These findings suggest a link between the gut microbiota-derived endotoxin and the pathogenesis of NAFLD, thereby evidencing a key role for the gut microbiota as an orchestrator of the gut-liver axis.

Taken together, there is compelling evidence that malmetabolism induced by intestinal microbiota dysbiosis is closely associated with the formation of fatty liver, fibrosis and liver cancer. This knowledge had led to growing interest in the intestinal microbiota as a new therapeutic target for the prevention and treatment of metabolic conditions including liver diseases [17–19]. Despite this considerable progress, the phylogenetic and functional compositions of gut microbiota associated with the liver disease progression and disease severity remain unclear. Understanding the link between the microbiota and the pathophysiology of liver diseases will help in the design of innovative therapies.

In this study, a streptozotocin-high fat diet (STZ-HFD) induced NASH-HCC C57BL/6J mouse model [20], which is highly relevant to human liver disease progression was prepared. Nearly 100% of mice in the model group follow disease progression from steatosis to NASH, fibrosis, and finally HCC, making this model well suited for profiling fecal microbial changes along with the liver disease progression. The aim of this study was to define the changes in the fecal microbiota over the entire disease spectrum in the NASH-HCC mouse model.

## RESULTS

### General information about the animal experiment

**Figure 1:**
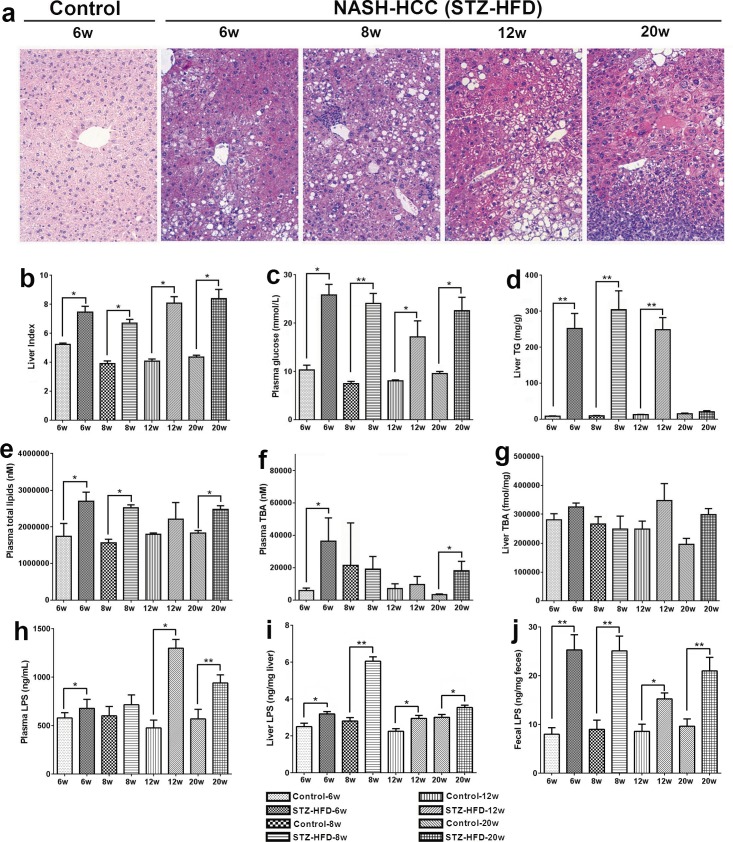
Pathophysiological features of NASH-HCC model mice **A.** H&E stained liver sections from control (week 6) mice and NASH-HCC mice at week 6, 8, 12 and 20. Original magnifications × 200. **B.** Liver index (ratio of liver to body weight (%)) at week 6, 8, 12 and 20. **C.** Fasting plasma glucose at week 6, 8, 12 and 20. **D.** Liver TG at week 6, 8, 12 and 20. **E.** Plasma total lipids level at week 6, 8, 12 and 20. **F.** Plasma total bile acids (TBA) at week 6, 8, 12 and 20. **G.** Liver total bile acids (TBA) at week 6, 8, 12 and 20. **H.** Plasma LPS level at week 6, 8, 12 and 20. **I.** Liver LPS level at week 6, 8, 12 and 20. **J.** Fecal LPS level at week 6, 8, 12 and 20. *, *p* < 0.05; **, *p* < 0.01, compared to controls.

Phenotypes of steatosis, NASH, fibrosis, and HCC were successfully developed in the STZ-HFD group. STZ-primed neonatal mice fed with HFD resulted in sequential histological changes from fatty liver (week 6), to NASH (week 8), fibrosis (week 12), and HCC at week 20 (Figure 1A). The liver index (ratio of liver to body weight) showed that all mice in STZ-HFD group had higher liver indices than the controls (Figure 1B). Fasting plasma glucose and liver TG were significantly higher in the STZ-HFD group compared to controls (Figure 1C and 1D). Total lipids were increased in plasma in STZ-HFD group than in controls (Figure 1E). The levels of total bile acids were increased in STZ-HFD group in steatosis, fibrosis and HCC phase except for NASH phase (Figure 1F and 1G). The LPS levels in plasma, liver and feces were markedly increased in STZ-HFD group compared to controls (Figure 1H, 1I and 1J and Figure 4).

### Over-all structural changes of gut microbiota in STZ-HFD treatment

To monitor shifts in the composition of fecal microbiota during development of NASH-HCC, high-throughput bar-coded pyrosequencing was performed. In total, 933,820 raw reads were generated and a total of 755 707 reads (average of 15743 ± 4907 S.D. reads per sample) were obtained for 48 samples after quality control. A total of 10 604 OTUs were then identified by grouping reads at the 97% similarity level. The Shannon diversity indices all reached stable values, indicating that bacterial diversity in these communities was mostly covered (Supplementary Figure S1A-D). The Rarefaction curves revealed that although new rare phylotypes would be expected with additional sequencing, most of the diversity had already been captured (Supplementary Figure S1E-H).

Compared with the controls, the STZ-HFD group exhibited lower alpha-diversity based on the number of observed species (*t* test, *P* = 0.005, 0.056, 0.006, and 0.003, respectively, at week 6, 8, 12 and 12). Other diversity indexes, including phylogeny-based metrics (PD Whole Tree) and Chao1 richness estimate were also measured. Results showed similar trends in comparative richness between groups. Compared with controls, STZ-HFD group exhibited significantly lower alpha-diversity as indicated by Chao1 (*t* test, *P* = 0.004, 0.004, 0.006, and 0.04, respectively) and PD Whole Tree (*t* test, *P* = 0.048, 0.10, 0.05, and 0.03, respectively) (Supplementary Figures S2A and S2B).

**Figure 2:**
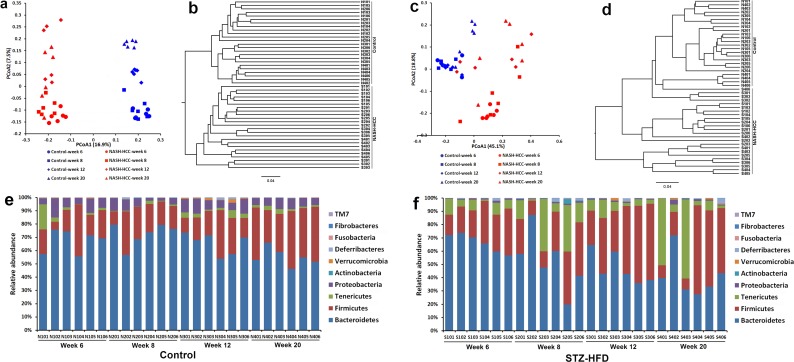
The overall gut microbiota structure change and taxonomic and functional variations in the mouse gut microbiota **A.** and **C.** A principal-coordinates (PCoA)-based characterization of overall community structure for mice from control group and STZ-HFD group at all-time points. QIIME was used to compute microbial β diversity with the unweighted (A) and weighted (C) UniFrac analysis. Sample similarities were projected onto two dimensions using principal coordinates. **B.** and **D.** Clustering of gut microbiota based on distances between the groups, calculated by multivariate analysis of variance tests of the first 48 PCs of unweighted (B) and weighted (D) UniFrac distance and conducted using DendroUPGMA. Cophenetic correlation coefficient value was 0.89 and 0.91, respectively. **E.** Relative abundance of major phyla across 48 fecal microbiota from mice in control. **F.** Relative abundance of major phyla across 48 fecal microbiota from mice in STZ-HFD group.

Beta diversity metrics also showed strong groupings of samples from the control and STZ-HFD group with statistically significant (*p* = 0.004). Unweighted PCoA revealed that the gut microbiota structure changed significantly in response to STZ-HFD treatment. STZ-HFD-related differences were mainly observed along the first principal coordinate (PCoA1), which accounted for the largest proportion (16.9%) of total variation (Figure 2A). This was confirmed by unweighted UniFrac Distance Matrix analysis, which first separated animals into two clusters corresponding to groups treated with or without STZ-HFD (Figure 2B). In all cases, clustering was supported by high cophenetic correlation coefficients (*r* = 0.89). UniFrac distances (a phylogenic-based, taxonomy-independent metric) between samples within one group were always smaller than any between-group comparisons, and PCoA of these distances showed that samples from each group clustered together (Figure 2). PCoA2, accounting for 7.5% of total variance, predominantly reflected age-related changes in gut microbiota composition. Weighted UniFrac PCoA was also used to discriminate the microbiota composition of the different groups, although no sharp separation was observed (Figure 2C), the largest variation was also explained by the treatment of STZ-HFD as revealed by weighted UniFrac Distance Matrix analysis (Figure 2D).

### SparCC-derived co-abundance network analysis

**Figure 3:**
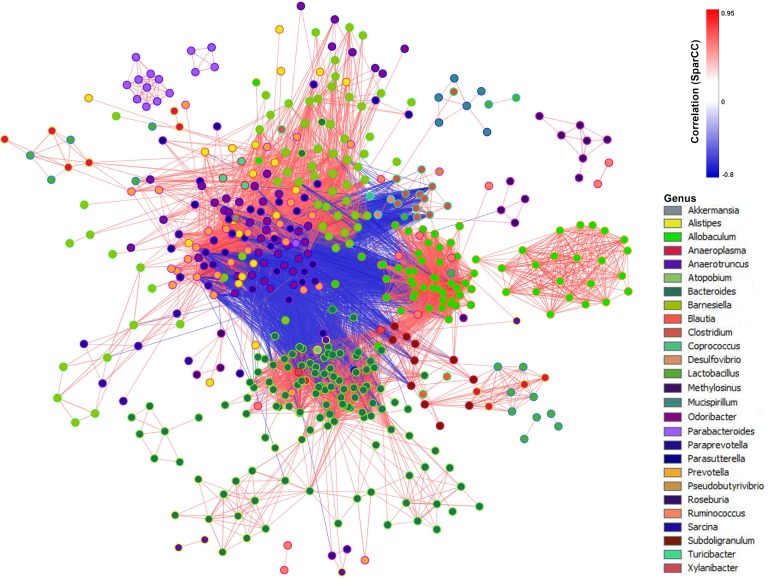
SparCC network plot of co-abundance and co-exclusion correlations between OTUs Nodes represent OTUs involved in either significant co-abundance (red edges) or co-exclusion (blue edges) relationships, with the magnitude of the correlation expressed as the intensity of the respective edge colors. The color of each node indicates the genus of the OTUs. Only significant correlations (two-sided pseudo p ≤ 0.05 based on bootstrapping of 100 repetitions) with an absolute correlation magnitude ≥0.6 are presented.

Extensive inter-species interactions exist in microbial communities [21]. Such interactions can thus be potentially reflected by the co-occurrence and co-exclusion patterns inferred from abundance profiles of phylotypes [22]. To obtain a measure of association between OTUs while incorporating their abundance with the progression of liver pathology, we inferred SparCC correlation coefficients using a recently described method that is robust for analyzing relative-abundance data [23]. We identified 15184 associations that had a p value less than 0.05; 11628 were positive (r ≥ 0.6) and 3556 were negative (r ≤ −0.6) from over 400,000 relationships were observed in total. We transformed the SparCC correlation measure into a network (Figure 3). The edge connecting each pair of nodes was the co-occurrence estimate inferred from the relative abundance profiles of species, which ranged from −0.768 to 0.941, suggesting strong co-exclusion and co-occurrence relationships between phylotypes.

### Key OTUs responding to STZ-HFD intervention

PLS-DA scores plot showed that different groups were clearly separated (Supplementary Figure S3). Based on the variable importance in the projection (VIP>1) and the *p* values from Mann-Whitney U test (*p* < 0.05), 378 OTUs responding to STZ-HFD treatment were identified (Supplementary Figure S4). OTUs belonging to *Bacteroides spp.* (19 OTUs), *Bacteroides vulgatus* (5 OTUs), *Atopobium spp.* (1 OTU), *Parabacteroides spp.* (1 OTU), *Xylanibacter spp.* (2 OTUs), *Lactobacillus spp.* (1 OTU), *Clostridium spp.* (1 OTU), *Sarcina spp.* (2 OTUs), *Dehalobacterium spp.* (1 OTU), *Pseudobutyrivibrio spp.* (2 OTUs), *Oscillibacter spp.* (1 OTU), *Oscillospira spp.* (1 OTU), *Ruminococcus spp.* (1 OTU), *Subdoligranulum spp.* (9 OTU), *Allobaculum spp.* (1 OTU), *Methylosinus spp.* (1 OTU), *Desulfovibrio spp.* (3 OTU), *Allobaculum sp id4* (1 OTU), *Clostridium cocleatum* (1 OTU) were significantly increased, whereas *Akkermansia spp.* (1 OTU), *Alistipes spp.* (29 OTUs), *Barnesiella spp.* (75 OTUs), *Blautia spp.* (1 OTU), *Lactobacillus spp.* (5 OTUs), *Odoribacter spp.* (73 OTUs), *Parabacteroides spp.* (2 OTUs), *Paraprevotella spp.* (21 OTUs), *Parasutterella spp.* (35 OTUs), *Prevotella spp.* (54 OTUs) were significantly decreased.

### Phylogenetic profiles of gut microbiota

Phylotypes with a median relative abundance larger than 0.01% of the total abundance in either the control group or the STZ-HFD group were included for comparison. Taxonomy-based analysis at the phylum level, Bacteroidetes and Firmicutes dominated the fecal microbial communities of both groups. Compared with the controls, the STZ-HFD mice had fewer Bacteroidetes and Proteobacteria, but higher levels of Firmicutes, Tenericutes and Actinobacteria (Figure 2E and 2F).

Taxonomy-based comparison at the genus level further showed that, *Bacteroides* was the dominant phylotype in both groups, but was significantly increased in STZ-HFD mice (Supplementary Figure S5). Of the remaining genera, *Roseburia, Subdoligranulum, Clostridium, Anaerotruncus, Oscillibacter, Mucispirillum, Coprobacillus, Xylanibacter, Pseudobutyrivibrio, Escherichia_shigella, Oscillospira, Peptococcus, Sarcina, Shuttleworthia, Papillibacter, Desulfovibrio, Atopobium, Anaerosporobacter*, and *Allobaculum* were enriched in STZ-HFD group, whereas *Barnesiella, Odoribacter, Parasutterella*, and *Moryella* were dominant in the control group.

**Figure 4:**
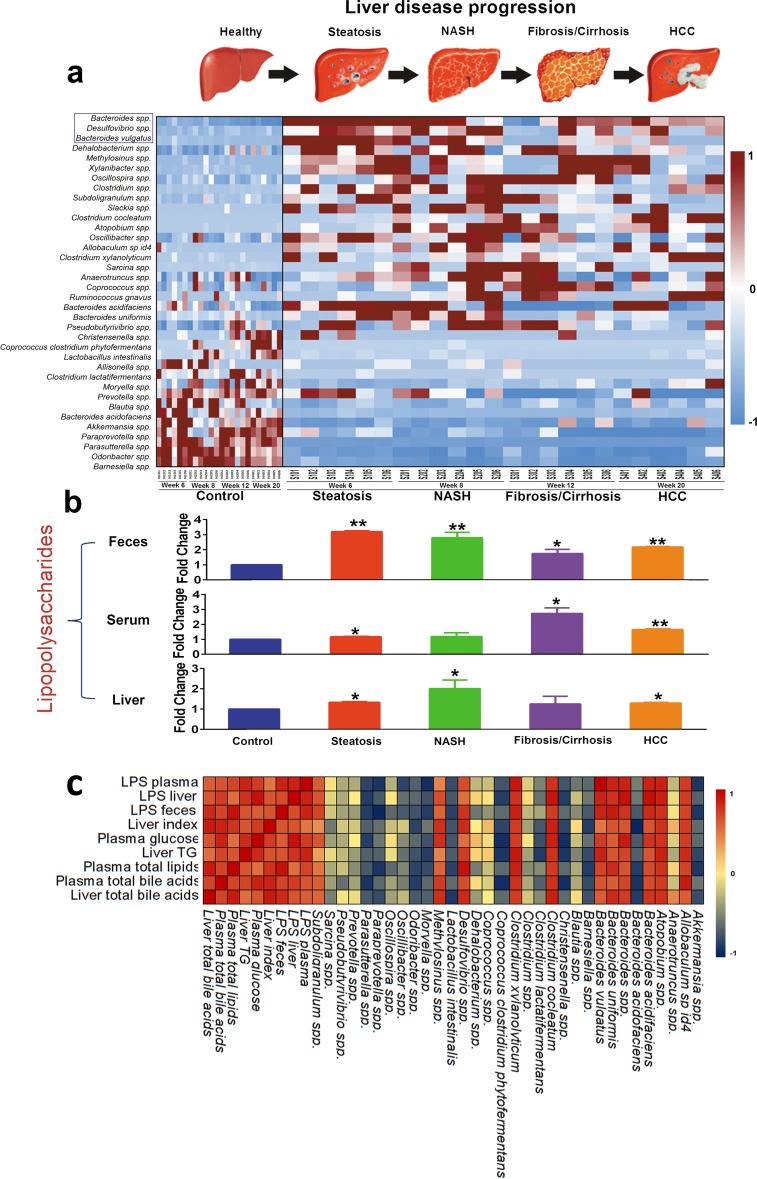
Gut microbiota changes are closely associated with the LPS, pathophysiological features, and commensurate with progression of liver pathology **A.** Heat map showing the relative abundance of major species for eight compact subgroups from week6, week 8, and week 12 to week 20. **B.** LPS levels in plasma, liver and feces were significantly increased. *, *p* < 0.05; **, *p* < 0.01, compared to controls. **C.** A correlation analysis was performed between gut microbiota and LPS and pathophysiological features, which revealed a range of correlation coefficients among the interactions between gut microbiota and LPS and pathophysiological features, ranging from 1.0 (maximum positive correlation) to −1.0 (maximum anticorrelation) and 0 (no correlation). Shades of dark red and dark blue represent positive correlation and negative correlation, respectively (see color bar scale).

The most abundant species in both control group and STZ-HFD group were primarily from the *Bacteroides* genus. The species *Bacteroides spp., Bacteroides vulgatus, Bacteroides uniformis, Clostridium spp., Clostridium xylanolyticum, Clostridium fusiformis cm973, Roseburia spp., Allobaculum sp id4, Subdoligranulum spp., Anaerotruncus spp., Oscillibacter spp., Xylanibacter spp., Mucispirillum schaedleri, Pseudobutyrivibrio spp., Desulfovibrio spp., Dehalobacterium spp., Oscillospira spp., Sarcina spp., Atopobium spp*., and *Peptococcus spp*. were increased in abundance in the STZ-HFD group. Of the species that decreased the most in abundance in the STZ-HFD group were *Parasutterella spp*., *Bacteroides acidofaciens, Odoribacter spp., Barnesiella spp., Moryella spp., Paraprevotella spp.*, *Lactobacillus intestinalis*, and *Akkermansia spp.* (Figure 4).

### Key phylotypes of the gut microbiota changes commensurate with progression of liver pathology

Our data demonstrated that the gut microbiota changed significantly in mice responding to STZ-HFD treatment (Figure 2 and Supplementary Figure S4). Those significantly altered gut microbiota were compared among STZ-HFD group at week 6, 8, 12 to week 20. For Gram-positive bacteria, the relative abundance of Firmicutes OTUs (%) in fecal samples was 25.05 at week 6, 26.47 at week 8 and increased to 44.20 at week 12 and then decreased to 34.89 at week 20 in STZ-HFD mice, whereas the relative abundance of Actinobacteria OTUs (%) were at very low level at week 6 (0.09), and then increased to 0.83 at week 8, 0.15 at week 12 and 0.20 at week 20. For Gram-negative bacteria, the relative abundance of Bacteroidetes OTUs (%) was decreased gradually from week 6 (66.48), week 8 (52.41), week 12 (47.40) to week 20 (41.16) and the relative abundance of Proteobacteria OTUs (%) was about the same level at week 6 to week 12 (0.85, 0.83, 0.80, respectively) but increased at week 20 (1.20). The Firmicutes/Bacteroidetes ratio was gradually increased from week 6 (0.39) to week 8 (0.717) to week 12 (1.034) to week 20 (1.011). Further analysis at the genus level revealed that the relative abundance of OTUs (%) of *Allobaculum* was gradually increased, whereas *Bacteroides* and *Paraprevotella* were gradually decreased along with the liver disease progression. The relative abundance of OTUs (%) of *Desulfovibrio* was significantly increased at week 8 compared to it at week 6 and remains the same level at week 12 and 20 as at week 6. The decreased abundance of *Bacteroides* was associated with a reduction in sequences assigned to *Bacteroides spp*. and *Bacteroides vulgatus*. The increased abundance of *Allobaculum* was associated with an increase in sequences assigned to *Allobaculum spp.* and the increase in *Desulfovibrio* was associated with an increase in sequences assigned to *Desulfovibrio spp*.

### Correlation between the gut microbiota and LPS and host pathophysiological features

Spearman correlation analysis between the gut microbiota change and LPS concentration in plasma, liver and feces and the host pathophysiological features listed in Figure 1 showed that *Akkermansia spp.*, *Christensenella spp.*, *Coprococcus clostridium phytofermentans*, *Lactobacillus intestinalis*, *Moryella spp.*, *Oscillibacter spp.*, *Paraprevotella spp.*, and *Parasutterella spp.* were significantly negatively correlated with LPS in plasma, liver and feces and pathophysiological features, and *Atopobium spp.*, *Bacteroides acidifaciens*, *Bacteroides spp.*, *Bacteroides uniformis*, *Bacteroides vulgatus*, *Clostridium cocleatum*, *Clostridium xylanolyticum*, and *Desulfovibrio spp.* were significantly positively correlated with LPS in plasma, liver and feces and pathophysiological features (Figure 4).

As most members in *Bacteroides* and *Desulfovibrio* were LPS-producers, the results showed that the genus population of *Bacteroides* and *Desulfovibrio* and the species population of *Bacteroides spp*., *Bacteroides vulgatus* and *Desulfovibrio spp.* were increased markedly when compared to the corresponding controls and STZ-HFD treatment significantly increased the LPS concentration (Figure 4 and Supplementary Figure S6).

## DISCUSSION

We have identified progressive alterations in gut microbiota associated with the development of liver disease. The alterations are characterized by significant increases in Firmicutes and Actinobacteria, and reduction in Bacteroidetes and Proteobacteria at phylum level. Significant alterations in gut microbiota were observed at the stage of liver steatosis and sustained throughout the spectrum of liver disease. These findings are consistent with the recent evidence that gut microbiota is a new crucial player in the complex chronic liver disease. Gut dysbiosis has been implicated in chronic metabolic disorders such as obesity, metabolic syndrome, diabetes, and cardiovascular diseases [24]. It is now also well established that gut microbiota and chronic liver disease are closely interrelated. Our study provides the first characterization of dynamic changes in gut microbiota during hepatocarcinogenesis in mice that mimics the human liver disease progression from steatosis, NASH, fibrosis and finally to HCC.

Chronic liver disease encompasses a spectrum of hepatic pathology. We observed that TG levels were significantly increased in fatty liver, NASH, and fibrosis phases but were markedly reduced in HCC. It has been shown that TGs accumulate in hepatocytes in the early stages of NAFLD and the accumulation of which may be a protective mechanism to prevent progressive liver damage [25]. The reduced levels of TG in liver at HCC phase is likely due to inadequate TG synthesis from fatty acids [26]. Lipid accumulation in the liver is the major hallmark of NAFLD [27], which is demonstrated with the increased total plasma lipid level responding to STZ-HFD treatment. HFD can increase body weight, liver weight and liver to body weight ratio in mice [28], which is consistent with our findings of the significantly increased liver index in STZ-HFD mice.

SparCC is a correlation methodology developed specifically for microbial data to eliminate the influence of compositional effects. SparCC network analysis suggests that not only members of the microbial communities, but also their co-abundance and co-exclusion relationships, were significantly altered in liver disease progression. The networks also highlight the potential importance of “minor” genera in the overall microbial interaction. Among the key phylotypes, the gut microbiota with positive or negative correlation with the LPS levels was similarly positively or negatively correlated with the pathophysiological features. The key phylotypes that were negatively correlated with LPS and pathophysiological features may include potentially beneficial bacteria that were found to be significantly decreased in model mice. Meanwhile, those key phylotypes that were positively correlated with LPS and pathophysiological features may comprise of potentially harmful bacteria that were significantly increased in model mice. Our study showed reduced microbial diversity and an increased ratio of Firmicutes to Bacteroidetes in the liver steatosis group, compared to the controls, which was maintained during the disease progression. Such a change at phylum level has been previously implicated in the pathogenesis of obesity in clinical and animal studies [29, 30]. We observed reductions in the abundance of gut barrier-protecting bacteria such as *Lactobacillus spp.* and increases in the abundance of Gram-negative endotoxin producing bacteria such as *Bacteroides spp*. and *Bacteroides vulgatus*, *Desulfovibrio spp*. It has been shown that such changes in the gut microbiota may increase intestinal permeability and circulating gut-originated antigens, primarily LPS [31–34]. Upon binding to the complex of CD14 and toll-like receptor 4 at the surface of innate immune cells, LPS can trigger the secretion of proinflammatory cytokines, which eventually impairs insulin sensitivity and induces insulin resistance-related metabolic disorders [31]. It was also reported that HFD will induce a leaky gut and more bacterial lysis, allowing the LPS of Gram-negative bacteria to enter the enterohepatic circulation [35]. In the present study, we measured the LPS levels in plasma, liver and feces, to investigate whether the LPS were changed due to the gut microbiota alteration responding to STZ-HFD treatment. In accordance with the results previously reported by Cani et al. [7], HFD induced a significant increase in plasma LPS levels in mice [31]. We also found a significantly negative correlation between *Akkermansia*, its level was significantly decreased in NASH-HCC model mice, and LPS in plasma, liver and feces. *Akkermansia* can reduce the LPS levels induced by HFD and increases in its abundance improves the metabolic profile of individuals with diet-induced obesity [36]. *Akkermansia* also has a role in reducing fat accumulation, its amount in the small intestine correlate negatively with the total body fat content [37]. We also observed reductions in *Parasutterella spp*. in model mice. This is consistent with a previous study which found significant decreases in *Parasutterella* in HFD-fed rats [38].

The study was designed to define the fecal microbiota changes over the entire disease spectrum with liver disease progression. However, there are limitations in the current study. First, we did not collect the small intestinal contents to analyze and investigate the changes of gut microbiota associated with liver disease progression. It was reported that there was bacterial overgrowth in the small intestine in precirrhotic liver diseases and cirrhosis [39, 40]. Further study on the small intestinal contents is needed. Second, we compared the fecal microbiota between control mice fed with normal diet and the model mice fed with STZ-HFD. However, HFD alone may result in a different microbiota profile between the groups and we did not perform comparison between control mice fed with HFD but without STZ and model mice fed with STZ-HFD to see the difference.

In summary, our work identified distinct and dynamic changes in gut microbiota in the pathological development of liver disease, providing important insights into host-microbe interactions in the development of liver disease and carcinogenesis. Targeted nourishment of these potentially beneficial bacteria that were negatively correlated with LPS and pathophysiological features and meanwhile, to inhibit those potentially harmful bacteria that were positively correlated with LPS and pathophysiological features may thus provide a promising strategy for the prevention and treatment of chronic liver disease and HCC.

## MATERIALS AND METHODS

### Animals and experimental design

Pathogen-free 14-day pregnant C57BL/6J mice were purchased from CLEA Japan (Tokyo, Japan) and maintained under specific pathogen-free conditions, on a 12-h light-dark cycle. New born male mice were divided into two groups: control group and NASH-HCC model group. Control mice were housed without any treatment and fed normal diet (CE-2 from CLEA, Japan, composed of 12 kcal% fat). The NASH-HCC mice were subjected to a single subcutaneous injection of 200 μg STZ (Sigma, MO, USA) 2 days after birth and feeding with HFD (HFD32 from CLEA, Japan, composed of 57 kcal% fat) *ad libitum* after 4 weeks of age for 16 weeks to induce a NASH-HCC [20]. The sample size used in this study was determined based on the expense of data collection, and the need to have sufficient statistical power. Body weights of all animals were recorded once a week. At week 6, 8, 12, and 20, 6 mice in each group were euthanized and their livers were removed and stored at −80°C for histological and lipid content analysis, including hematoxylin-eosin (H&E) staining and triglyceride (TG) analysis (see Supplementary Information). Fasting blood glucose was measured using an automatic biochemical analyzer (Hitachi 7180, Tokyo, Japan). Plasma, liver and fecal LPS concentrations were determined using a mouse LPS Elisa kit (BlueGene Biotech, Shanghai, China, see Supplementary Information). Plasma total lipids and total bile acids levels were also measured. Mice body weight and liver weight were recorded. Liver index was calculated as the ratio of liver to body weight. Before sacrifice, fecal samples were collected from each mouse. All stool samples were stored at −80°C prior to 16S rDNA gene sequencing.

All animal procedures were performed in accordance with the ‘‘Guide for the Care and Use of Laboratory Animals’’ prepared by the National Academy of Sciences and published by the National Institutes of Health (NIH publication 86-23, revised 1985).

### Gut microbiota characterization

Genomic DNA was extracted from each fecal sample as described previously [32, 41, 42]. A bacterial tag-encoded FLX 16S rDNA amplicon pyrosequencing approach (bTEFAP, MR DNA, www.mrdnalab.com) was used to target the V1-V3 hypervariable region as previously reported [43]. 16S universal Eubacterial primers 27Fmod (AGRGTTTGATCMTGGCTCAG) and 519Rmod (GTNTTACNGCGGCKGCTG) containing barcodes unique to each sample were used. A single-step PCR using HotStarTaq Plus Master Mix Kit (Qiagen, Valencia, CA) were used under the following conditions: 94°C for 3 minutes, followed by 28 cycles of 94°C for 30 seconds; 53°C for 40 seconds and 72°C for 1 minute; after which a final elongation step at 72°C for 5 minutes was performed. Following PCR, all amplicon products from different samples were mixed in equal concentrations and purified using Agencourt Ampure beads (Agencourt Bioscience Corporation, MA, USA). Samples were subject to DNA pool emulsion PCR and sequenced utilizing Roche 454 FLX titanium instruments and reagents and following manufacturer's guidelines.

The Q25 sequence data derived from the sequencing process was processed using a proprietary analysis pipeline (www.mrdnalab.com, MR DNA, Shallowater, TX). The data was processed with the Usearch program in the QIIME pipeline [44]. The pyrosequencing chimeras were discarded using the Uchime filtering also in the QIIME pipeline. Sequences are depleted of singletons, short sequences < 200bp, sequences with ambiguous base calls, and sequences with homopolymer runs exceeding 6bp. Sequences are then denoised and chimeras removed. The resulting filtered reads were binned according to barcode after which adapter, barcode, and primer sequences were removed. Reads were clustered into operational taxonomic units (OUTs) were defined with clustering at 3% divergence (97% sequence similarity) [43, 45, 46]. OTUs were then taxonomically classified using BLASTn against a curated GreenGenes database [47, 48] and compiled into each taxonomic level into both “counts” and “percentage” files. Counts files contain the actual number of sequences while the percent files contain the relative (proportion) percentage of sequences within each sample that map to the designated taxonomic classification. For example, if there are 1000 sequences and 100 of the sequences are classified as *Clostridium* then we represent this as *Clostridium* being 10%.

SparCC (available at https://bitbucket.org/yonatanf/sparcc) was employed to represent co-abundance and co-exclusion networks between OTUs. SparCC and calculation of two-sided pseudo p values (p values ≤ 0.05 considered significant) were run on python scripts based on bootstrapping of 100 repetitions. A network plot was generated, and correlation magnitudes ≥ 0.6 (indicating strong co-abundance relationships) and ≤ − 0.6 (indicating strong co-exclusion relationships) were plotted. Visualization of the network was achieved using Cytoscape v3.2.1.

Heatmap and hierarchical clustering analysis of the OTUs were performed using the pheatmap package v1.0.7 running in R v3.2.1 (http://www.r-project.org).

### Data analysis

Rarefaction analysis, Phylogenetic Diversity (PD) Whole Tree, Chao1 richness estimate and the Shannon diversity index were calculated using Qiime [44]. The phylogenetic tree was then used for both weighted and unweighted UniFrac principal coordinates analysis (PCoA) [49]. Cluster analysis was conducted using DendroUPGMA [50]. Partial least squares- discriminant analysis (PLS-DA) was used to test whether these groups could be separated based on the OTU data [38]. All multivariate statistical analyses were performed with SIMCA-P+ 13.0 (Umetrics, Umeå, Sweden). All other statistical analyses were calculated using GraphPad Prism (version 6.0; GraphPad Software, San Diego, USA) and SPSS 22.0 (IBM SPSS, USA). Data are expressed as mean ± SEM. To test difference between the groups in biochemical measurements for statistical significance, normally distributed data were analyzed by tests with Holm-Sidak method for multiple comparisons correction. Data that did not meet the assumptions of analysis were analyzed by the Mann-Whitney U test. We regarded *p* values of < 0.05 as significant. Spearman correlation analysis was made to evaluate the interactions between gut microbiota and LPS levels in plasma, liver and feces, and pathophysiological features, giving a value ranging from 1.0 (maximum positive correlation) to −1 (maximum anticorrelation) and 0 (no correlation).

## SUPPLEMENTARY MATERIALS FIGURES


