# Supplementary material for: Distinctly altered gut microbiota in the progression of liver disease
Source: Oncotarget. 2016 Mar 29;7(15):19355–66. doi: 10.18632/oncotarget.8466 (PMC4991388; doi:10.18632/oncotarget.8466)
Supplement: Supplementary file 1 [file oncotarget-07-19355-s001.pdf]

Distinctly altered gut microbiota in the progression of liver disease

Supplementary Materials

Supplementary Methods

Liver histological analysis

Liver tissues were fixed in 10% neutral-buffered formalin, embedded in paraffin blocks, and processed by routine hematoxylin and eosin (H&E) staining. The stained sections were subsequently examined for histopathological changes.

Measurement of lipid triglyceride in the liver

The triglyceride (TG) in the liver will be measured using the TG Colorimetric Assay Kit ((Cayman, Ann Arbor, MI) according to the kit protocol. Briefly, accurately weighted liver (~10 mg) were homogenized with 0.3 mL of the diluted Standard Diluent containing protease inhibitors using a homogenizer (Next Advance, Averill Park, NY) for 3 min. Finally, the homogenate was centrifuged at 10 000 × g for 10 minutes at 4°C and the supernatant was used for the TG assay.

LPS analysis in blood plasma, liver and feces samples

Plasma, liver and fecal LPS concentrations were determined using a mouse LPS Elisa kit (BlueGene Biotech, Shanghai, China) according to the manufacturer's protocol.

For blood plasma samples, frozen plasma samples were thawed on ice and vortexed and then 100 µl of each sample and a series of diluted standards were assayed following the steps in the manufacturer's protocol.

For feces samples, accurately weighted feces (~30 mg) were extracted with water and supernatant was subjected to LPS assay.

For liver tissues, accurately weighted liver (~30 mg) were homogenized with certain amount of PBS using a homogenizer (Next Advance, Averill Park, NY) for 3 min. Finally, the homogenate was centrifuged at 10 000 × g for 10 minutes at 4°C and the supernatant was used for the LPS assay.

All of the samples were tested in duplicate, and optical density values were measured using a kinetic microplate reader (VMax, Molecular Devices, CA, USA) at a wavelength of 405 nm.

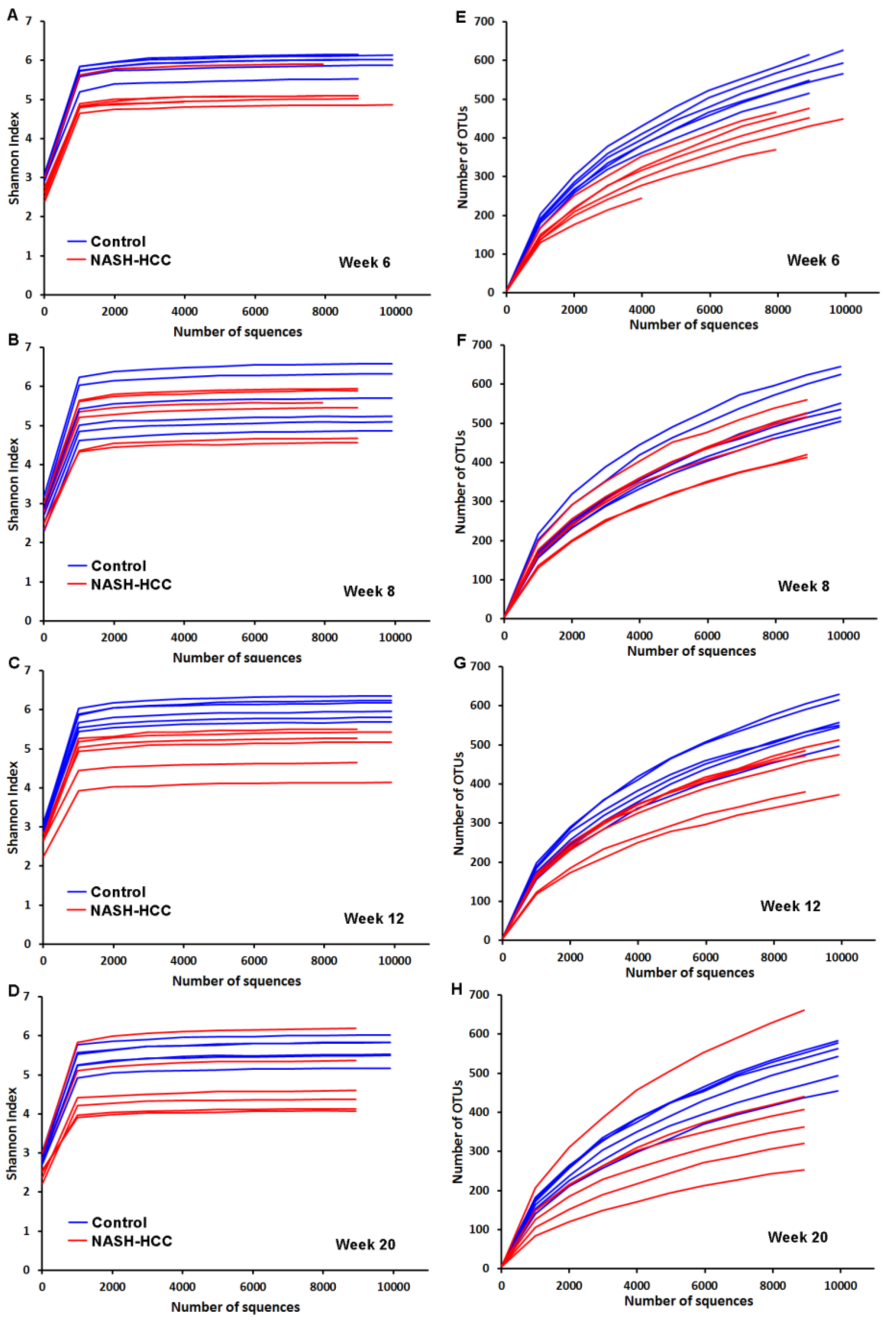

Figure S1. Evaluation of the sequencing depth in each sample. (A–D) Shannon diversity index curves of the samples at weeks 6, 8, 12 and 20. (E–H) Rarefaction curves of the samples at weeks 6, 8, 12 and 20.

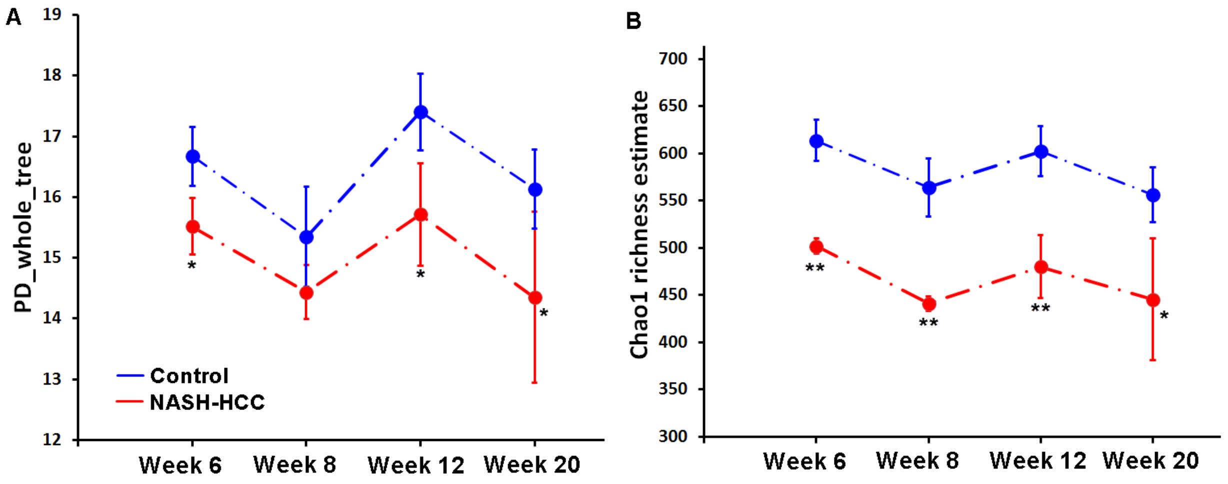

Figure S2. Richness and diversity of the gut microbiota. (A) OTU estimates via rarefaction analysis. (B) Chao1 richness estimates. Calculations were performed after rarefying an equal number of sequence reads for all samples. Values are expressed as means ± standard error. QIIME was used to compute microbial β diversity with the weighted UniFrac analysis. *, $p<0.05$; **, $p<0.01$, when compared to controls.

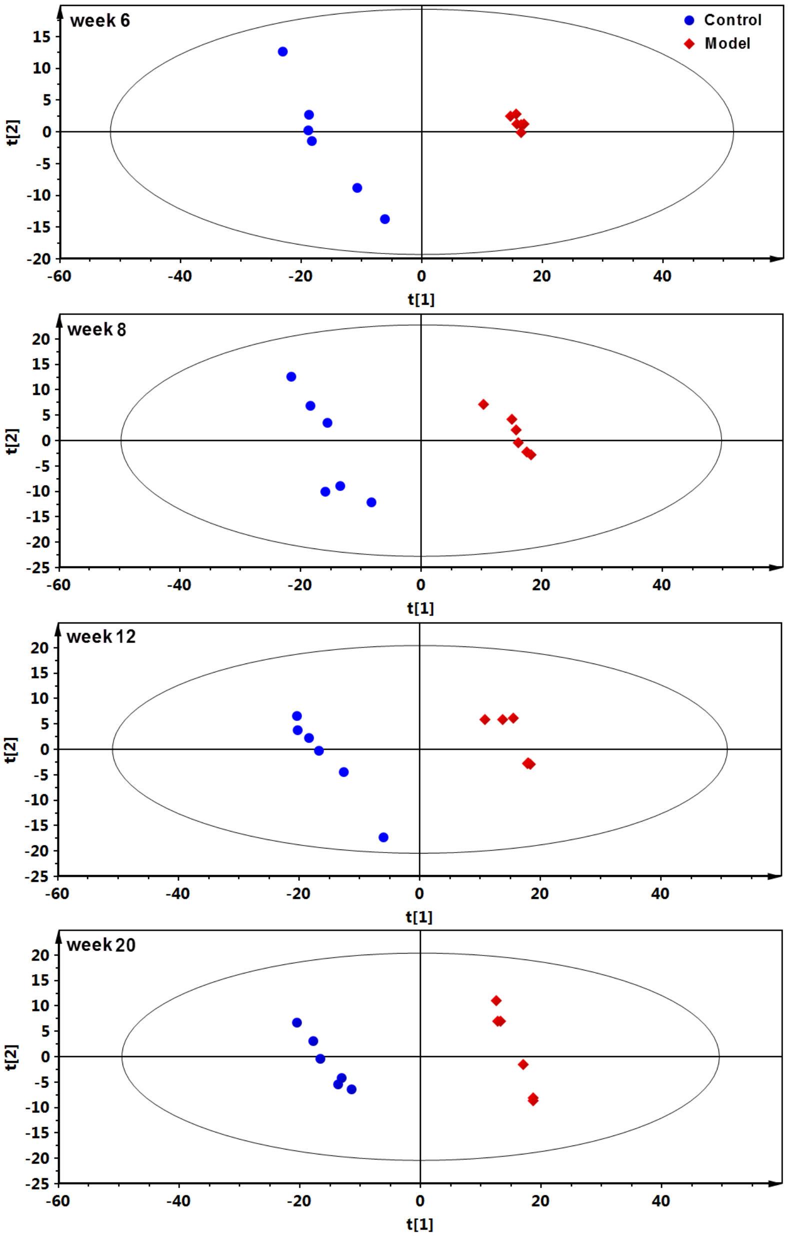

R2X = 0.596, R2Y = 0.995, Q2(cum) = 0.93

R2X = 0.67, R2Y = 0.999, Q2(cum) = 0.974

R2X = 0.59, R2Y = 0.995, Q2(cum) = 0.937

R2X = 0.565, R2Y = 0.996, Q2(cum) = 0.974

Figure S3. Partial least squares-discriminant analysis (PLS-DA) identified the differential gut microbiota compositions accountable for the separation between groups at OTUs level.

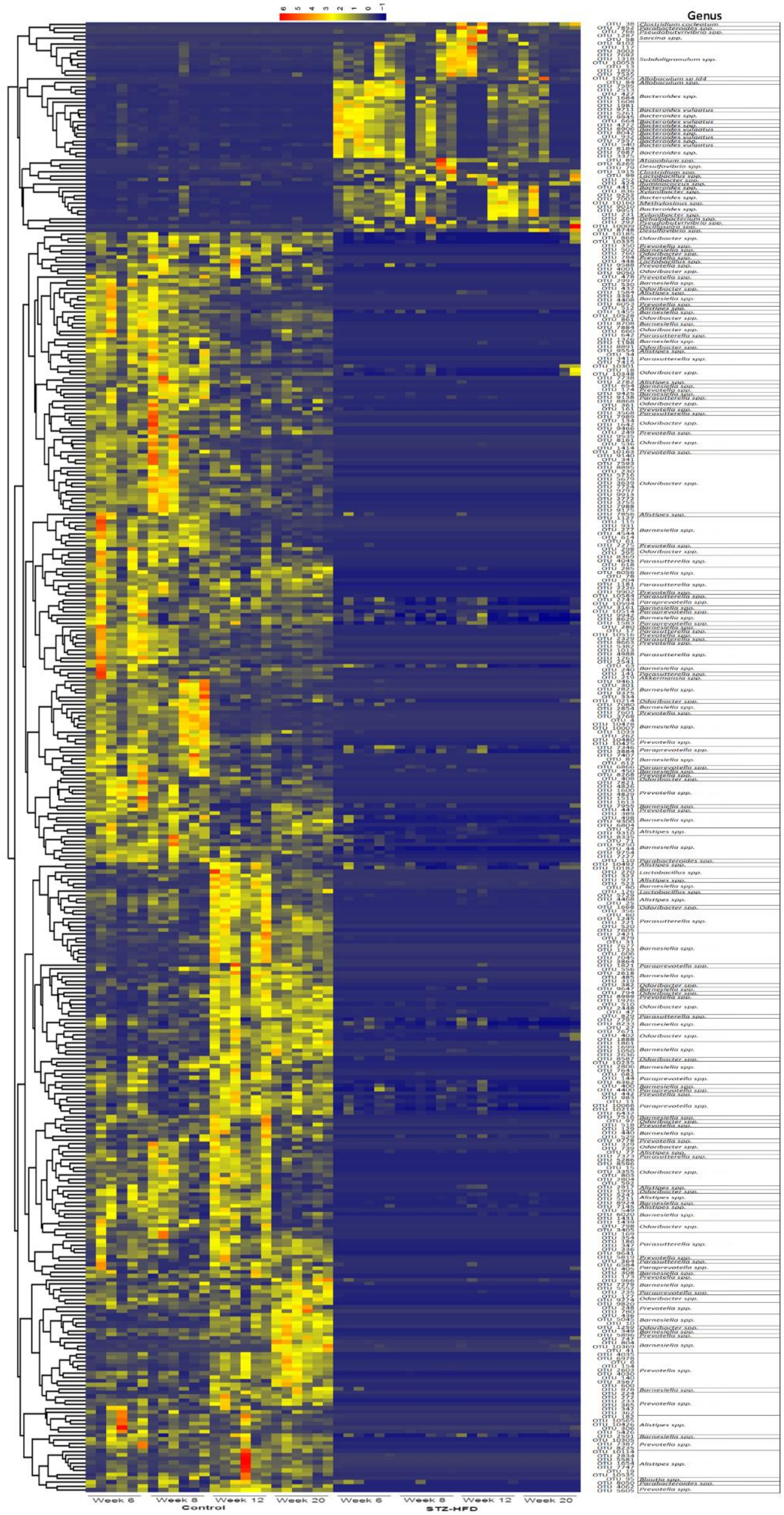

6 5 4 3 2 1 0 −1
Genus
Week 6
Week 8
Week 12
Week 20
Control
Week 6
Week 8
Week 12
Week 20
STZ-HFD

Figure S4. Heatmap of key OTUs responding to STZ-HFD treatment. Partial least squares discriminant analysis together with Mann Whitney U test was used to identify the key OTUs. The color of the spot corresponds to the Z-score normalized relative abundance of OUT. The OTUs are organized according to their phylogenetic relationships. The genus names of the OTUs are shown on the right.

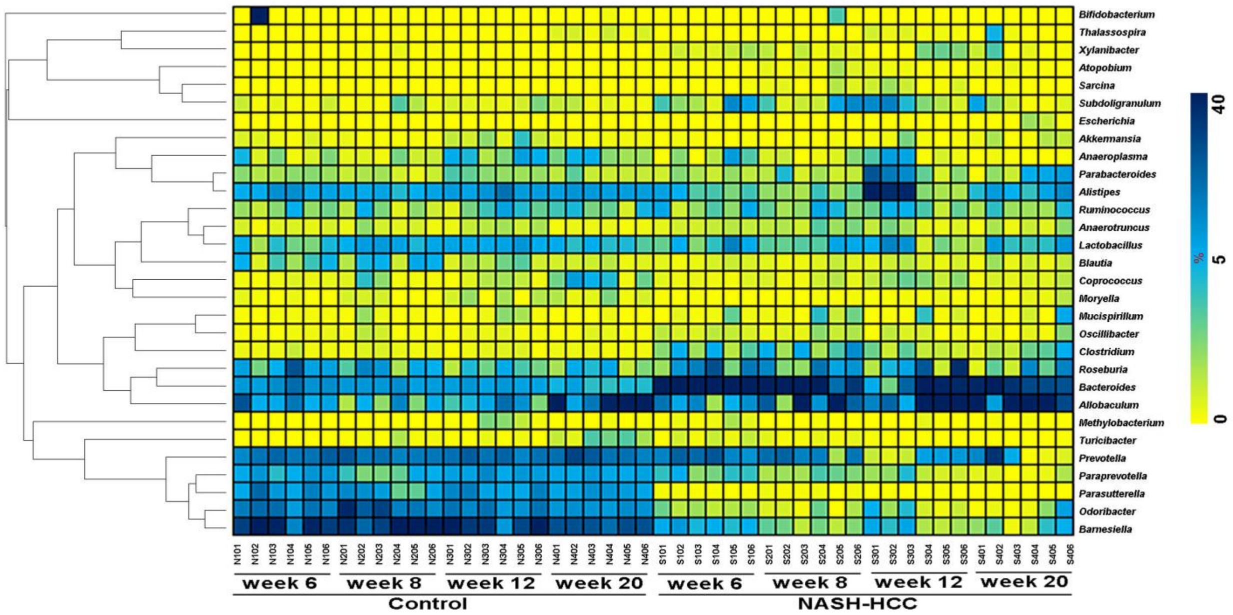

Figure S5. **Heatmap of major genera in control and STZ-HFD group**. Heat map showing the relative abundance of major genera (contributing >1% of the total abundance in at least one sample) for eight compact subgroups. The phytogenetic relationships of these bacteria are displayed to the left of the heatmap, with animals arranged as control group and NASH-HCC model group from week6, week 8, and week 12 to week 20.

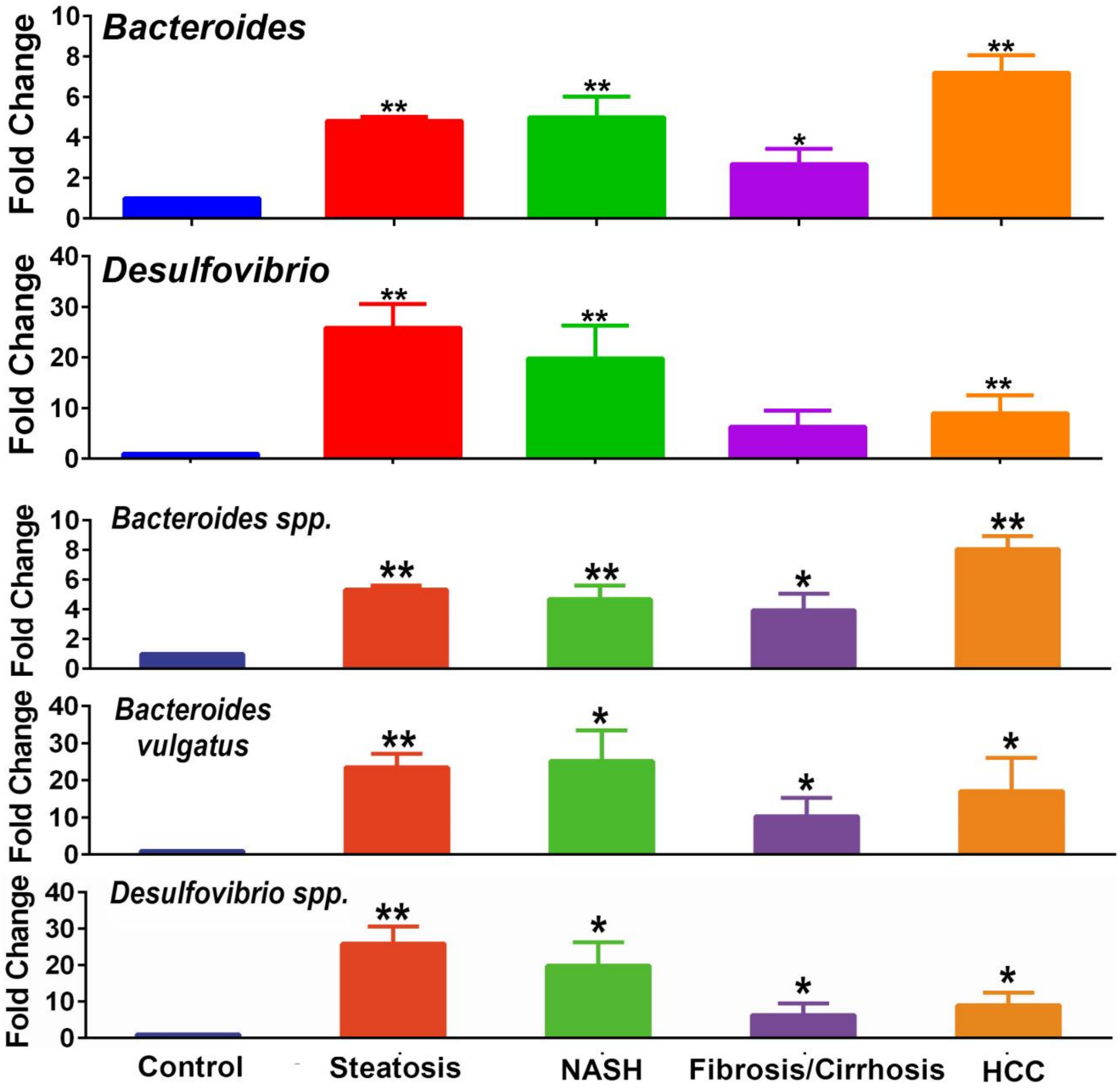

Figure S6. The genus population of *Bacteroides* and *Desulfovibrio* were increased markedly in model mice as compared to the corresponding controls at week 6, 8, 12 and 20. *, $p < 0.05$; **, $p < 0.01$, compared to controls.
